# Impact of Protein N^α^-Modifications on Cellular Functions and Human Health

**DOI:** 10.3390/life13071613

**Published:** 2023-07-24

**Authors:** Yie-Hwa Chang

**Affiliations:** Edward A. Doisy Department of Biochemistry and Molecular Biology, Saint Louis University Medical School, Saint Louis, MO 63104, USA; yiehwa.chang@health.slu.edu

**Keywords:** protein modification, methionine aminopeptidases, acetylation, myristoylation, methylation, ubiquitin modification, actin modification, cancer, Parkinson’s disease

## Abstract

Most human proteins are modified by enzymes that act on the α-amino group of a newly synthesized polypeptide. Methionine aminopeptidases can remove the initiator methionine and expose the second amino acid for further modification by enzymes responsible for myristoylation, acetylation, methylation, or other chemical reactions. Specific acetyltransferases can also modify the initiator methionine and sometimes the acetylated methionine can be removed, followed by further modifications. These modifications at the protein N-termini play critical roles in cellular protein localization, protein-protein interaction, protein-DNA interaction, and protein stability. Consequently, the dysregulation of these modifications could significantly change the development and progression status of certain human diseases. The focus of this review is to highlight recent progress in our understanding of the roles of these modifications in regulating protein functions and how these enzymes have been used as potential novel therapeutic targets for various human diseases.

## 1. Introduction

**Figure 1:**
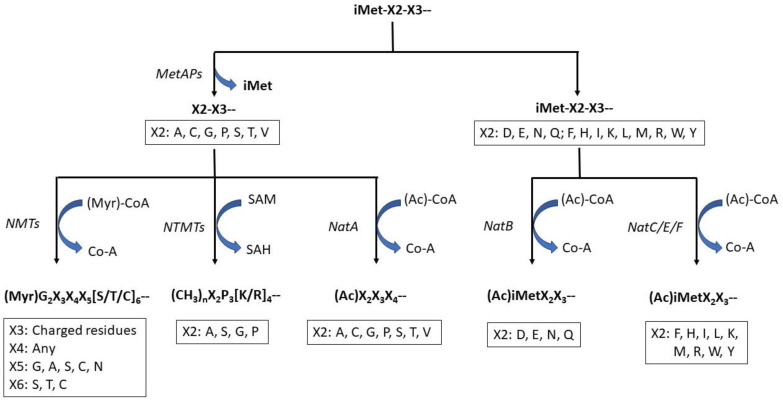
Summary of N^α^-modifications of Cytosolic Human Proteins. Methionine aminopeptidases (MetAPs) are responsible for N-terminal iMet excision (NME) [1,2]; N-terminal acetyltransferases (NATs) for N^α^-acetylation [3]; N-terminal myristoyltransferase (NMTs) for N^α^-myristoylation [4]; and N-terminal methylation for N^α^-methylation (NTMTs) [5].

In the cytosol of human cells, when a newly synthesized polypeptide emerges from the ribosomes, its fate can be determined by the enzymes that modify its N-terminal α-amino acid residue (N^α^). These N-terminal modifications include excision of the initiator methionine (iMet), N^α^-myristoylation, N^α^-acetylation, N^α^-methylation, and other less common modification events such as N^α^-propionylation, N^α^-palmitoylation, N^α^-arginylation, and N^α^-ubiquitylation (Figure 1). Among these enzymes, methionine aminopeptidases (MetAPs) are responsible for N-terminal iMet excision (NME) [1,2]; N-terminal acetyltransferases (NATs) for N^α^-acetylation [3]; N-terminal myristoyltransferase (NMTs) for N^α^-myristoylation [4]; N-terminal methylation for N^α^-methylation (NTMTs) [5]; N-terminal palmitoylacyltransferases (PATs) for N^α^-palmitoylation [6]; and ubiquitin ligases for ubiquitylation of the N-terminal α-amino acid residue [7]. The NATs can also sometimes catalyze a much less understood modification: N^α^-propionylation [8]. These modifications of proteins at their N-termini play critical roles in many important cellular processes, and dysregulation of these events could significantly impact the development and progression of certain human diseases [9,10]. The focus of this review is to highlight recent progress in our understanding of the substrate specificity of these enzymes, their roles in the biological function of specific proteins, how they might be regulated, the crosstalk between different modifications, and how these enzymes are used as targets for potential novel therapeutical strategies.

## 2. N-Terminal Methionine Excision (NME)

Protein synthesis in the cytosol of a eukaryotic cell, in most cases, is initiated with methionine. When the second amino acid residue is a small and uncharged amino acid such as Ala, Cys, Gly, Pro, Ser, Thr, or Val, iMet is usually removed co-translationally by two types of methionine aminopeptidases (MetAPs) [11,12,13,14,15,16,17,18,19,20]. Although these two types do not share a high sequence identity, their catalytic domains belong to the same family of metalloproteases with a typical “pita-bread” protease fold [19]. The N-terminal domain of eukaryotic MetAP1s contains two zinc finger motifs; a RING-finger-like Cys2-Cys2 zinc finger and a Cys2-His2 zinc finger related to RNA-binding zinc fingers [21,22]. These two zinc fingers are essential for the regular functional association of MetAP1 with the ribosomes [21,22]. Eukaryotic type 2 MetAPs (MetAP2s), on the other hand, contain an N-terminal domain with a positively charged Lys-rich region [16,17,18,19,20]. Deleting MetAP1 in yeast leads to a slow growth phenotype which can be rescued by overexpressing MetAP2, whereas knocking out both MetAPs is lethal, indicating that the NME process is vital for normal cell growth (Table 1) [17]. This finding strongly suggests that both enzymes act on similar groups of substrates in vivo. Structural studies of human MetAPs revealed a potential difference in the substrate specificity of their catalytic sites due to more steric restrictions in MetAP1 [20]. Proteomics analysis of the substrate specificity of human MetAPs indicates that MetAP2 prefers iMet-Val and iMet-Thr. However, substrate specificity is significantly overlapping for human MetAP1 and MetAP2 [15].

Since discovering that human MetAP2, not human MetAP1, is the molecular target of TNP-470, a potent anti-angiogenesis inhibitor, MetAP2 has become a drug target for treating cancer, obesity, Prader-Willi Syndrome (PWS), and autoimmunity (Table 2) [23,24,25,26]. Inhibitors for human MetAP2 are well tolerated in patients at therapeutically relevant doses and have been developed for a variety of pharmaceutical applications, including the treatment of cancer [27,28,29,30,31], diabetes, and obesity [32], as well as the modulation of autoimmunity [33,34]. Although none of these inhibitors have yet passed Phase III clinical trials, the interest of the drug development community remains high due to continued promising preclinical and clinical efficacy results for novel MetAP2 inhibitors. Unfortunately, most studies did not assess the impact of MetAP2 inhibition on cellular functions, making it harder to correlate the phenotypes to the inhibitors’ mode of action. Most of time, more questions were raised than answered regarding the role of MetAP2 in these diseases after a new clinical study. For example, it is still being determined whether the molecular mechanisms driving each phenotype discovered during each clinical trial share the exact molecular mechanisms. The molecular mechanisms of MetAP2 inhibitor-induced weight loss or immune modulation remain to be established. Even the fundamental questions regarding the substrate specificity of MetAPs in different tissues still need to be better defined. Indeed, a better understanding of MetAP biology and the mode of action of MetAP2 inhibitors would undoubtedly improve the quality of biomarkers for patient screening, the identification of novel indications, and the development of evidence-based drug combinations in targeted disease treatment.

In the mitochondria of human cells, protein synthesis is initiated with formyl-methionine. A deformylase can remove the formyl group to expose an unmodified methionine, which becomes a substrate for MetAP. A search of the GenBank database with cytosolic MetAP1 and MetAP2 protein sequences led to the discovery of MetAP1D [59]. MetAP1D is a new member of the human MetAP family and belongs to the Type I MetAP subfamily. Phylogenetic analysis of human MetAP isoforms suggests that human MetAP1D pairs with mitochondrial MetAP orthologs previously identified in plants [90]. All three MetAP isoforms can remove Met from a Met-Ala-Ser peptide in vitro. However, the substrate specificity of MetAP1D has not been fully investigated. MetAP1D is overexpressed in colon cancer cells and colon tumors. Reduced expression of MetAP1D by shRNA has been shown to decrease the ability of colon cancer cells to grow in soft agar, indicating that overexpression of MetAP1D may be necessary for tumorigenesis. Thus, MAP1D has been suggested to be a target for chemotherapy in colon carcinoma [59,60]. 

Recently, genomic analyses demonstrated that patients with intellectual disability (ID) harbor a novel homozygous nonsense mutation in the *MetAP1* gene. ID is a common genetic and clinically heterogeneous disease, and underlying molecular pathogenesis can frequently be unidentified by whole-exome/genome testing. Improper neuronal function from losing essential proteins could lead to neurologic impairment and ID [91]. In addition, a mutation in the *MetAP1D* gene was identified as one candidate involved in the penetrance of Leber’s hereditary optic neuropathy (LHON) [92]. Though we are still very early in understanding how mutations in MetAPs could affect human health, NME excision processes provide a promising avenue in translational research.

## 3. N^α^-Myristoylation

N-myristoyltransferase (NMTs) are responsible for protein N^α^-myristoylation. NMTs can transfer a C:14:0 acyl-CoA to the N-terminal glycine (Gly) of specific groups of proteins following the excision of iMet by MetAPs [93,94]. N-terminal Gly is absolutely required for NMT activity, with preferred substrates containing sequence: G_2_X_3_X_4_X_5_(C/S/T)_6_K_7_ [95,96,97,98] in which X_3_ favors a charged residue; X_4_ can be any residue; X_5_ favors Gly, Ala, Ser, Cys or Asn; X_6_ favors Cys, Ser, or Thr; whereas Trp, Phe, Tyr, and Pro are prohibited [97,98,99,100]; and K_7_ interacts with negatively charged residues in the binding pocket of MNTs [97,98,99,100]. The substrate specificity of MNTs might be regulated by their interacting partners in different cells and species [100]. There are variations in sequence preferences across species [96,98,100]. A tool predicting species-specific N^α^-myristoylation before experimental data is available will help develop NMT-targeted therapy. 

Most human tissues express 2 NMT isoenzymes (HsNMT1 and HsNMT2) [101,102,103,104,105]. These 2 isozymes share ~77% protein sequence identity. Although they share similar substrates, they are not considered functionally redundant [101,102,103,104,105,106,107,108,109]. At least 40 NMT substrates have been discovered in human cells. These proteins are usually inserted into the lipid rafts, plasma membrane, endoplasmic reticulum (ER), Golgi apparatus, nuclear membrane, or mitochondria in cells [109,110,111,112,113,114]. Thus, depending on the subcellular localization of the myristoylated protein, it can regulate diverse cellular functions [35,36,37,38,61], including signal transduction [36,37,38], cellular transformation [36,37,38], oncogenesis, both innate and adaptive immune responses, cancer, and human immunodeficiency virus (HIV) infection [61], as well as parasitic and fungal diseases [62,63]. Small-molecule NMT inhibitors have therapeutic potential in viral and parasitic infections and cancer. For example, IMP-366 (DDD85646), a bioactive pyrazole sulfonamide inhibitor of *Trypanosoma brucei* NMT with an apparent Ki value of 1.44 nM, is a widely used NMT inhibitor that can suppress picornavirus replication, as well as malaria and sleeping sickness parasites by inhibiting their NMTs [64,65,66]. IMP366 can also suppress breast and colon cancer cell growth [67]. PCLX-001 (DDD86481) is a potent, small molecule inhibitor of both human NMTs. Preclinical studies have shown that PCLX-001 markedly inhibits hematologic and lymphoma cell lines in tissue culture and achieves complete remissions in human cancers grown in immunodeficient mice [68] and tumor responses in solid cancers [68,69,70,115]. It can nullify N^α^-myristoylation of Src family kinases and promote their degradation, leading to cancer cell death in vitro and xenograft models [68]. The molecule has been extensively investigated in non-clinical safety testing [70] and found suitable for formal drug development in humans. Recently, analysis of pharmacokinetic and pharmacodynamic endpoints revealed that PCLX-001 has pharmacokinetic properties suitable for continued development as an oral, once daily, cancer therapy. A more in-depth understanding of structural differences between human and pathogenic NMTs and human NMT1 and NMT2 will further advance the development of small molecules with increased selectivity and decreased toxicity.

## 4. N^α^-Acetylation

**Figure 2:**
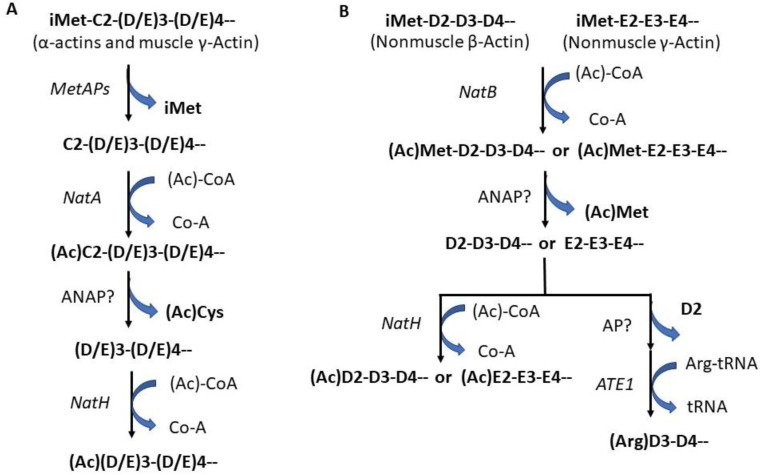
N^α^-modifications of actins. (**A**) Muscle γ-actin and α-actins, typically containing a Cys in the second position followed by charged amino acid residues. After the iMet is removed by MetAPs, the second Cys, likely by NatA, is acetylated and the acetylated Cys will be further removed by ANAP to expose the acidic residue in the third position [136]. This newly exposed acidic residue will be further acetylated post-translationally by NatH. (**B**) Nonmuscle β-actin and γ-actin contain a string of negatively charged residues following iMet, MDDD in β-actin, and MEEE in γ-actin. The iMet is acetylated by NatB and then possibly removed by an unidentified actin N-acetyl-aminopeptidase (ANAP) [136]. Like its cytoplasmic partner γ-actin as discussed in Section 4, the iMet of β-actin acetylated by NatB, and then possibly removed by an unidentified ANAP is co-translationally. Next, the exposed second residue (Asp2 in β-actin, Glu2 in γ-actin) will be further acetylated by a dedicated N-acetyltransferase, NatH/NAA80 [141,142,143]. However, some β-actin N^α^-termini will not be acetylated, instead they undergo further proteolytic processing, and the new N^α^-termini (DD-) are then N^α^-arginylated by ATE1. N^α^-acetylation or N^α^-arginylation of actins will change their N-terminal charge density and affect actin structure and function.

N-terminal acetylome analysis revealed that ~80–90% of the N-terminal α-amino group of soluble human proteins is acetylated. At least nine different N-terminal acetyltransferases (NATs) are involved in this modification event in which acetyl-CoA is used as a cofactor for the chemical reaction (Figure 1) [115,116]. This modification replaces the positive charge associated with the free α-amino group with a polar group and can block it for further changes. Each NAT has different substrate specificities [117,118], which can affect critical protein functions, including complex formation [39,40,41,42], protein localization [43,44,45], and protein degradation governed by the N-end rules (Table 1) [46,47,48]. NATs usually contain a unique catalytic subunit and one or two auxiliary subunits. The auxiliary subunits play various roles, including ribosomal anchoring [119,120]. In humans, 7 NATs have been identified: NatA (NAA10 and NAA15), NatB (NAA20 and NAA25), NatC (NAA 30, NAA 35, and NAA38), NatD (NAA40), NatE, NatF, and NatH (NAA80) [121,122,123]. NatA, NatB, and NatC are responsible for most protein N^α^-acetylation [3]. NatA and NatB are heterodimeric complexes, each containing a specific catalytic subunit and a unique auxiliary subunit (NAA10 in NatA and NAA20 in NatB, as the catalytic subunit; NAA15 in NatA and NAA25 in NatB as the auxiliary subunit) [124]. NatC is a heterotrimeric complex.I It contains 1 catalytic subunit (NAA30) and 2 auxiliary subunits (NAA35 and NAA38) [125]. NatA, NatB, and NatC each have different substrate specificity. NatA acetylates small N-terminal residues after iMet excision [115], whereas NatB acetylates the iMet with sequences of MD- ME-, MN-, and MQ- [126,127]. NatC/E/F have overlapping substrates, acting on iMet when followed by residues that are not D, E, N, and Q [116,127,128,129,130,131]. When an additional catalytic subunit (NAA50) binds to NatA, NatE is formed as a dual enzyme complex with crosstalk between 2 catalytic subunits, NAA10 and NAA50 [71,121]. NatF binds to the Golgi membrane and acetylated transmembrane proteins [71,132,133]. NatD contains only a single catalytic unit, NAA40, with no auxiliary subunit. It acetylates the α-amino group of Ser of histones H4 and H2A after the exciton of iMet [134,135]. NatH targets all six mammalian actin isoforms in unique processing steps that differentiate muscle and non-muscle actins. Non-muscle actins contain a string of negatively charged residues following iMet, MDDD in β-actin, and MEEE in γ-actin. The iMet is acetylated by NatB and then removed by an unidentified actin N-acetyl-aminopeptidase (ANAP) [136]. Muscle actins, typically containing a Cys in the second position, are first processed by MetAPs to remove the iMet, followed by acetylation of the second Cys, likely by NatA, and the acetylated Cys will be further removed by ANAP to expose the acidic residue in the third position [136]. In all cases, this newly exposed acidic residue will be further acetylated post-translationally by NatH (Figure 2) [133]. Knockout of the *NatH* gene results in an increased filamentous to globular actin ratio, increased filopodia and lamellipodia formation, and accelerated cell motility [133]. 

Moreover, many studies have demonstrated that NatB and NatD have connections to various human diseases [71,72,73,74,75,76,77,78,79,80,81,137,138,139,140]. For example, NatB is upregulated in human hepatocellular carcinoma, and silencing NatB mRNA can block cell proliferation and tumor formation [139]. Thus, NatB could be a potential therapeutic target for certain cancers [139]. N^α^-acetylation of α-Syn by NatB in human cells can increase its stability and lipid binding and reduce aggregation capacity [74,75,76,77,78]. Since α-Syn is a critical protein in Parkinson’s disease, NatB might play a role in PD pathogenesis (Table 2) [72,73]. A recent report on the Cro-EM structure of human NatB complexed with a CoA-α-Syn conjugate provided new insights into the mode of substrate selection of NAT enzymes, which will further facilitate the development of small molecule NatB probes [71]. NatD, on the other hand, plays essential roles in a diverse range of tumors, and its expression level correlates with poor survival of cancer patients [71,79]. However, unlike NatB, NatD is downregulated in hepatocellular carcinoma tissues, and ectopic NatD expression sensitizes hepatoma cancer cell lines to drug-induced apoptosis [54,71]. Moreover, a recent study has indicated that NatD is a critical regulator of cell invasion during lung cancer metastasis [139]. Interestingly, in colorectal cancer (CRC) cells, NatD plays a pro-survival role suggesting that it may stimulate cancer cell growth [71.81]. Despite the exciting discoveries of NatD’s essential roles in cancer development and metastasis, it remains elusive regarding its role in cancer chemotherapy response.

## 5. N^α^-Methylation

N-terminal methyltransferases catalyze protein N-terminal methylation (N^α^-methylation). These enzymes are conserved between yeast (Tae1) and humans (NTMT1 and NTMT2). They catalyze the transfer of the methyl group from S-adenylmethionine (SAM) to the free α-amino group of the newly exposed X2 residue of a nascent polypeptide after iMet excision that contains a sequence motif, iMet-X2-P3-[K/R]4, in which X2 is A, S, G, or P (Figure 1). This motif is recognized as the canonical N-terminal motif for NTMTs [49,50]. The properties of N^α^-methylated proteins differ according to the degree of methylation. Adding one methyl group to the α-amino group only slightly increases its basicity and introduces a minor steric hindrance that may slightly reduce its reactivity. However, adding two or three methyl groups can generate a permanent positive charge in the α-amino group. Until now, no eraser of this event has been identified, and protein N^α^-methylation is considered irreversible. The two human NTMTs target various substrates associated with diverse biological pathways (Table 1) [50,51,52,53,54,55,56,57,58]. N^α^-methylation regulates protein–protein and protein–DNA interactions [49,50,51]. For example, N^α^-methylation (trimethylation) of CENP-A is critical for its formation of the centromere complex with two other partners, CENP-I and CENP-T, which is essential for cell cycle progress and cell survival [56,57]. On the other hand, the loss of N^α^-trimethylation of CENP-B prevents it from binding to the centromeric DNA motif [56,57,58]. In HEK293T cells, N^α^-methylation of DDB2 promotes its nuclear localization to UV light-induced cyclobutane pyrimidine dimer (CPD) foci and stimulates CPD repair, suggesting N^α^-methylation’s protective role against UV damage [82,83]. N^α^-methylation of MYL9, a transcriptional activator of intercellular adhesion molecule 1 (ICAM1), weakens its interaction with an actin-modulating protein, Cofilin-1, and promotes ICAM1 transcription [83]. N^α^-methylation of RCC1 can regulate the RCC1-chromatin interaction by inhibiting the association of its core portion with histones H2A or H2B [84]. The binding of Ran to N^α^-methylated RCC1 triggers the exposure of its histone-binding surface and promotes the interaction between the N^α^-methylated RCC1 tail and negatively charged DNA [84,85,86]. This electrostatic interaction is N^α^-methylation dependent. The loss of N^α^-methylation reduces RCC1 binding to DNA and causes mitotic defects [85]. Interestingly, N^α^-methylation of MRG15 was recently found to be modulated by m^6^A writers, leading to new regulation of N^α^-methylation by the m^6^A-based epitranscriptome [86]. In summary, increasing evidence indicates that N^α^-methylation is essential in regulating mitosis, chromatin interactions, DNA repair, tRNA transport, and maintaining genome stability (Table 1) [82,83,84,85,86,87,88,89]. Dysregulation of NTMTs has been implicated in the pathogenesis of various diseases, including breast, colorectal, pancreatic, and lung cancers (Table 2) [144,145]. 

## 6. Other N^α^-Modifications

### 6.1. N^α^-Palmitoylation

Unlike N^α^-myristoylation, much less is known about N^α^-palmitoylation. Protein palmitoylation usually occurs at an internal Cys residue [146], but researchers have recently identified several N^α^-palmitoylated proteins. For example, a palmitoyl group is found to be attached to the α-amino group of the N-terminal Gly residue of the α-subunit of the heterotrimeric G protein that is responsible for the activation of adenylyl cyclase [146,147]. In addition, the secreted vertebrate signaling proteins Hedgehog (Hh) and Sonic Hedgehog (Shh) are N^α^-palmitoylated at the Cys residue after the cleavage of the N-terminal signal sequence [148,149]. Hedgehog protein acyltransferase (Hhat) is suggested to be responsible for palmitoylating Shh [150,151,152,153]. This modification regulates Shh signaling strength [150,151,152,153,154,155]. That belongs to the family of transmembrane proteins termed MBOAT (membrane-bound O-acyltransferase) [154] which acylates Shh during its passage through the secretory pathway [150].

### 6.2. N^α^-Ubiquitylation

Protein ubiquitylation usually refers to the addition of ubiquitin to the ε-amino group of an internal Lys residue via a combined activity of ubiquitin-activating (E1), conjugating (E2), and ligating (E3) enzymes. However, protein N^α^-ubiquitylation refers to adding ubiquitin to the newly exposed α-amino group of a protein. In both cases, the ubiquityl group may serve as a target for polyubiquitylation, a well-known degradation signal recognized by the proteasome [156,157]. N^α^-ubiquitylation was first discovered by Ciechanover’s lab [158]. However, the first direct evidence revealed by MS analysis was the N^α^-ubiquitylation of HPV-58 oncoprotein E7 [7,159]. As HPV-58 E7 contains no lysine residues, its degradation is likely solely dependent upon N^α^-ubiquitylation. Recently, α-synuclein and a tau tetra repeat domain were found to be N^α^-ubiquitylated in vitro. N^α^-ubiquitylation affected its aggregation properties and was proposed to enable targeting of the modified α-synuclein, and a tau tetra repeat domain to the proteasome for degradation, suggesting a role of N^α^-ubiquitylation in removing amyloidogenic proteins [160]. A total of 2 enzymes, E3 HUWE1 and E2 Ube2w, catalyze N^α^-ubiquitylation [161,162,163]. HUWE1 was shown to ubiquitylate the N-terminus of a MyoD mutant that contains no Lysine residues [163]. Ube2w, on the other hand, can successfully ubiquitylate the N-terminus of a lysine-free version of Ataxin-3 and Tau [161]. There are some distinctive differences when comparing the active site of Ube2w to that of classical E2s. The unique structure features of Ube2w make its novel active site more suitable to accommodate an α-amino group rather than a Lys side chain [161,162]. Interestingly, a recent report found that Pro in positions 2 to 4 of an unstructured peptide backbone has an inhibitory effect on Ube2w activity [164]. We are still very early in studying the biochemistry and biology of protein N^α^-ubiquitylation.

### 6.3. N^α^-Arginylation

**Figure 3:**
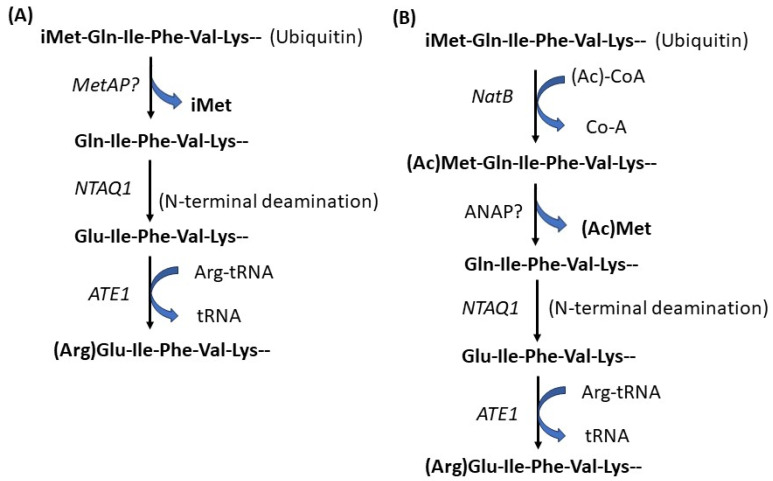
N^α^-modifications of ubiquitin. (**A**) Ubiquitin (Ub) starts with Met-Gln-Ile (MQ) N-terminus. N^α^-modifications of ubiquitin involve NME possibly by an unknown MetAP, N-terminal deamination by NTAQ1 N-terminal Gln amidase, and N-terminal arginylation by ATE1 arginyltransferase [187,188,189] ubiquitin might be processed by the NME-provoked cascade reactions of N-terminal deamination and N-terminal arginylation to yield RE-Ub (Figure 3) [186]. (**B**) However, according to the specificity of NatB, iMet is likely to be acetylated by NatB. If so, the acetylated iMet will be removed by an unknown deacetylase. Then, the newly exposed Gln is converted to Glu by NTAQ1 N-terminal Gln amidase, followed by N^α^-arginylation by ATE1.

Protein arginylation was discovered in 1963 [165,166]. The enzyme arginyltransferase (ATE1) responsible for this modification was first cloned and characterized in yeast [167]. ATE1 catalyzes the transfer of Arg from aminoacyl-tRNA to target proteins post-translationally [167]. The *ATE1* gene exists in nearly all eukaryotes. The *ATE1* gene encodes four isoforms in humans and mice, generated by alternative splicing [168,169]. ATE1 preferentially targets the unacetylated acidic N-terminal residues, including Asp and Glu [170]. It has also been found to target oxidized Cys [171,172] at a far lesser frequency and targeting oxidized Cys was found to differ between different ATE1 isoforms [173,174]. Recent high throughput analysis of arginylation suggested the existence of a consensus motif that may potentially be used to predict arginylation sites in vivo [173,174]. ATE1-mediated N^α^-arginylation has been initially characterized as part of the N-degron (N-end rule) pathway that regulates the protein’s half-life [175,176]. N-terminally arginylated proteins can be recognized by specific E3 ligases of the ubiquitin–proteasome system (UPS) to ubiquitinate a nearby Lys for the follow-up degradation. However, if there is no accessible Lys for the E3 ligases, the N-terminally arginylated proteins will remain metabolically stable. Calreticulin [177] is one of ATE1 target. N^α^-arginylation of calreticulin is less susceptible to proteasomal degradation than the non-arginylated form [178], and the modification triggers its translocation from the ER into the cytosol, increasing apoptotic response [179]. Actin is another known target of ATE1. N^α^-arginylation or N^α^-acetylation of cytoplasmic β-actin is emerging as a first-line mechanism to regulate cell migration [180,181,182]. Like its cytoplasmic partner γ-actin as discussed in Section 4, the iMet of β-actin acetylated by NatB, and then removed by an unidentified ANAP is co-translationally. Next, the exposed second residue (Asp2 in β-actin, Glu2 in γ-actin) will be further acetylated by a dedicated N-acetyltransferase, NatH/NAA80 [141,142,143]. The acetylated Asp2 or Glu2 can be removed, and then the Asp3 or Glu 3 can be arginylated by the nonspecific arginyltransferase Ate1 (Figure 2) [173,183]. Arginylation of γ-actin with slower translation leads to its immediate proteasomal degradation [184]. However, arginylated β-actin (R-actin) has been shown to specifically relocate to the leading edge upon induction of cell migration (Figure 2) [181]. *NatH* knockout (KO) cells show an increase in R-actin level by seven-fold [173,184,185], which supports the hypothesis that acetylation and arginylation of β-actin are mutually exclusive and that the increased level of R-actin could be an essential factor for the enhanced motility of *NatH* KO cells. However, it remains unclear whether the impact of N^α^-modification on actin directly changes its interactions with the associated proteins or indirectly affects its interaction with the associated proteins via altered intrinsic interactions between actin monomers within the actin filament [185]. Recently, Nguyen KT et al. found that ubiquitin is a target of Ate1 by identifying the arginylated ubiquitin (RE-Ub) in yeast [186]. Ubiquitin (Ub) starts with Met-Gln-Ile (MQ) N-terminus. The authors proposed that N-terminal modifications of mammalian ubiquitin based on their new findings involve NME by unknown MetAP, N-terminal deamination by NTAQ1 N-terminal Gln amidase, and N-terminal arginylation by ATE1 arginyltransferase [187,188,189] ubiquitin might be processed by the NME-provoked cascade reactions of N-terminal deamination and N-terminal arginylation to yield RE-Ub (Figure 3) [186]. However, according to the specificity of NatB, iMet is likely to be acetylated. If so, the acetylated iMet will be removed by an unknown deacetylase. Then, the newly exposed Gln is converted to Glu by NTAQ1 N-terminal Gln amidase, followed by N^α^-arginylation by ATE1 (Figure 3). Further studies of this exciting N-terminal modification of selected targets will eventually unravel the full complexity of the N-terminal arginylome and the biological significance of this event. 

## 7. Regulation and Crosstalk

The substrate specificity and catalytic efficiency of MetAP2, but not MetAP1, are regulated by an allosteric disulfide bond, Cys228-Cys448, located at the rim of the active site. Oxidized and reduced isoforms of MetAP2 have different catalytic activities on their peptide substrates [190]. When solid tumor cells adapt to a limiting blood supply, they experience different degrees of stress, such as hypoxia, glucose deprivation, and growth factor. This cellular stress can lead to increased production of reactive oxygen species that may lead to an increased level of oxidized MetAP2 with altered substrate specificity [190]. Since excision of iMet is a prerequisite for specific N^α^-modifications such as N^α^-myristoylation, N^α^-methylation, and N^α^-acetylation events via NatA, altered MetAP activity could indirectly affect the downstream N^α^-modifications. N^α^-modifications, except the abovementioned possible indirect regulation by MetAP activity, are generally considered static because no specific modification eraser(s) have been discovered yet. Many recent reports indicate that dynamic crosstalk occurs between N^α^-methylation and other modifications, including N^α^-acetylation, internal methylation on lysine or arginine, phosphorylation, and m^6^A modifications in RNA [79]. Knockdown of *NatD* reduced metastasis and invasion of lung cancer cells. The proposed mechanism was that N^α^-acetylation of histone H4 antagonizes the CK2α-mediated phosphorylation on the same serine residue (H4S1ph) to regulate Slug expression and metastasis [79]. The differential impact of N^α^-methylation and N^α^-acetylation on the subcellular localization of MYL9 is the first report on the interplay between methylation and acetylation at the same site [89,191,192]. Both N^α^-methylation and Ser phosphorylation on the N-terminal tail of RCC1 were concurrent during mitosis [134,193]. In asynchronous HeLa cells, S1 phosphorylation decreased by about 25% in the RCC1 N^α^ methylation-defective mutant compared with wild-type RCC1, suggesting that N^α^-methylation has a positive effect on the phosphorylation of S1 [134]. In mitotic cells, no significant change was observed in the total phosphorylation levels of two Ser residues (S1 and S10) regardless of N^α^-methylation. However, the phosphorylation level of S2 increased by 10% in the absence of N^α^-methylation [134]. Furthermore, a recent study demonstrated that NTMT1 protein expression might be regulated by readers, writers, and erasers involved in the N^6^-methyladenosine (m^6^A) modification of mRNA, providing a critical first piece of evidence for the regulation of N^α^-methylation [134,193], which introduces additional dimensions that govern the interplay among different modifications at the N^α^-position. 

## 8. Conclusions

Our understanding of protein N^α^-modifications has been significantly advanced in the past decades. Protein N^α^-modifications are critical in controlling protein/protein interaction, protein-DNA interaction, cellular protein localization, and protein stability. The dysfunction or dysregulation of the enzymes involved in N^α^-modification has been connected to various human diseases, including cancer, neurodegenerative diseases, and infectious diseases. Therefore, many of these enzymes have been used as targets for developing novel therapeutic approaches for disease treatment, including cancer, diabetes, and obesity. We can anticipate that scientists using cutting-edge technologies such as cryo-EM and multi-omics approaches will make significant advances in this exciting field in the future.

## Figures and Tables

**Table 1:** Impact of protein N^α^-modifications on cellular functions.

Enzymes	Cellular Functions	References
MetAP1	Cell cycle progression, cell proliferation, enzyme function, protein stability, cellular localization	[13,14,15,16,17,18,19,20,21,22,23]
MetAP2	Angiogenesis, B-cell differentiation, cell specific Cytotoxicity	[23,24,25,26,27,28,29,30,31,32,33,34]
NMTs	Signal transduction, cellular transformation, innate immune responses, adaptative immune response	[35,36,37,38]
NATs	Actin cytoskeleton structure, cell cycle progression, cell proliferation cell mobility	[39,40,41,42,43,44,45,46,47,48]
NTMTs	Protein stability, protein-protein interaction, protein-DNA interaction, cellular localization, response to cellular stress, DNA repair, regulation of mitosis, chromatin interaction, tRNA transport, genome stability	[5,49,50,51,52,53,54,55,56,57,58]

**Table 2:** Potential targets for developing a novel treatment for human diseases.

Enzymes	Targeted Human Diseases	References
MetAP1	Antibiotics, cancer	[20]
MetAP1D	Colon cancer	[59,60]
MetAP2	Cancer, obesity, diabetes, Prader-Willi syndrome, autoimmunity	[23,24,25,26,27,28,29,30,31,32,33,34]
NMTs	Cancer; HIV, fungal, and parasitic infection	[61,62,63,64,65,66,67,68,69,70]
NATs	Cancer (lung, liver, colon), Parkinson’s disease	[71,72,73,74,75,76,77,78,79,80,81]
NTMTs	Cancer (breast, colon, pancreatic, lung)	[82,83,84,85,86,87,88,89]

## Data Availability

No new data were created.
